# Detecting Splicing Variants in Idiopathic Pulmonary Fibrosis from Non-Differentially Expressed Genes

**DOI:** 10.1371/journal.pone.0068352

**Published:** 2013-07-02

**Authors:** Nan Deng, Cecilia G. Sanchez, Joseph A. Lasky, Dongxiao Zhu

**Affiliations:** 1 Department of Computer Science, Wayne State University, Detroit, Michigan, United States of America; 2 Tulane Cancer Center, School of Medicine, Tulane University, New Orleans, Louisiana, United States of America; Centro Cardiologico Monzino IRCCS, Italy

## Abstract

Idiopathic pulmonary fibrosis (IPF) is an interstitial lung disease of unknown cause that lacks a proven therapy for altering its high mortality rate. Microarrays have been employed to investigate the pathogenesis of IPF, but are presented mostly at the gene-expression level due to technologic limitations. In as much as, alternative RNA splicing isoforms are increasingly identified as potential regulators of human diseases, including IPF, we propose a new approach with the capacity to detect splicing variants using RNA-seq data. We conducted a joint analysis of differential expression and differential splicing on annotated human genes and isoforms, and identified 122 non-differentially expressed genes with a high degree of “switch” between major and minor isoforms. Three cases with variant mechanisms for alternative splicing were validated using qRT-PCR, among the group of genes in which expression was not significantly changed at the gene level. We also identified 35 novel transcripts that were unique to the fibrotic lungs using exon-exon junction evidence, and selected a representative for qRT-PCR validation. The results of our study are likely to provide new insight into the pathogenesis of pulmonary fibrosis and may eventuate in new treatment targets.

## Introduction

Idiopathic pulmonary fibrosis (IPF) is a progressive form of chronic lung scarring, which occurs predominantly in older adults and carries a dismal prognosis. Studies indicate that 50% of patients with IPF die within 3 years of diagnosis [1] and that the majority of afflicted patients die from IPF [2]. To date there are no known agents that reduce mortality of IPF and clinical trials are stymied by a dearth of clinically employed biomarkers. Our understanding of the pathogenesis of IPF is far from complete, and to date there has been a lack of powerful, high throughput molecular profiling techniques that permit delineation of the whole transcriptome landscape at high resolution.

The human transcriptome is much larger than the human genome on account of alternative RNA splicing. According to the most recent Ensembl database of human alternative splicing and transcript diversity (ASTD), there are more than 100,000 annotated human transcripts encoded by about 20,000 human genes. It is estimated that between 50% and 70% or even more of human genes have at least one alternative splice variant [3]–[5]. The set of required transcripts and corresponding proteins within a cell or organ varies as a function of the stage of development and changing environmental conditions, such as wound repair, and alternate splicing is a significant means of modulating the protein set [6], [7].

It has been proposed that SR protein-dependent exon skipping may constitute a strategy for establishing tissue-specific alternative splicing critical for cell differentiation programs [8]. SR proteins are Serine-Arginine–rich proteins, which bind to specific RNA domains and are critical factors for the splicing process. Among these, SF2/ASF has been reported to act as a splicing enhancer [9], [10]. SF2/ASF function is regulated by phosphorylation of the RS domain, which affects both protein–protein interactions and is necessary for splicing [11], [12]. A hallmark pathologic feature of IPF is the fibroblastic foci, which consist of clusters of myofibroblasts and the connective tissue matrix they deposit [13]. SF2/ASF modulates fibronectin splicing which has been shown to be crucial for myofibroblast differentiation [14], [15]. Activation of the Akt/mTOR pathway during myofibroblast differentiation directly influences alternative FN splicing through enhancement of SF2/ASF phosphorylation [15], [16].

Splice variants occur in conjunction with fibrosis in the lung and other organs. For example, a distinctive feature of cellular fibronectin in IPF is the inclusion of at least one of two extra exons, termed Extra Type III Domain A (EDA) and Extra Type III Domain B (EDB), which arise on account of alternative exon splicing. The EDA splice variant of fibronectin is up-regulated in IPF and locates to the site of fibrogenesis [17], and thus has potential as a biomarker. A tenascin C splice variant is expressed in both pulmonary fibrosis, including in our human IPF samples, and cirrhosis [18], and may serve as a biomarker for cirrhosis [18], [19]. As another example, IL-4 is a profibrotic TH2 cytokine. The IL-4δ2 splice variant, with a deletion in exon 2, acts as a dominant negative. The ILδ-4 splice variant is increased in the airways of asthmatics and asthmatic airways undergo fibrosis [20]. Therefore, splicing variants are present in fibrotic lung and could potentially be used to identify active fibrogenesis.

Traditional methods use high throughput gene expression profiling techniques, such as microarray, to detect differentially expressed genes at the whole-transcriptome scale. In-depth examination of the splicing of the top ranked genes using lower throughput, but more accurate techniques, such as qRT-PCR [21], can be subsequently performed. These approaches have proven useful, but they do not permit a comprehensive transcriptomic landscape at the level of splicing variants.

The declining cost and increasing throughput of RNA-seq technology provide new opportunities to characterize the highly diverse and complex human transcriptome. Compared with the older tilting and exon arrays, RNA-seq provides abundant signal at base-pair resolution, and promises a better means to identify and quantify splicing variants in the human transcriptome [22]–[26]. Examining transcriptomes at the isoform-level allows for detecting differential regulated splicing variants encoded by the non-differentially expressed genes, which may be important but are often hidden from discovery by many older microarray techniques. Recent years have seen a plethora of new computational methods, such as [27]–[30], for detection of differential splicing using RNA-seq. These methods are effective in detecting differential splicing events. Here we apply our method and report the whole transcriptome-scale analysis of differential splicing events in IPF patient samples using RNA-seq. To the best of our knowledge, this is the first study that examines splicing variants from non-differentially expressed genes for IPF. We verify a few of the most consistently differential splicing events using qRT-PCR and discuss some mechanistic insights.

## Results

### Quality Check and Alignment Rate of RNA-seq Data

We confirmed the high short read quality of each sample, with the average quality score at each base position above 35 using fastQC, which is much higher than the recommended threshold of 20. For each sample, there are more than 26 million 54-mer reads. There were between 60% and 70% of reads uniquely aligned to the reference genome, representing in a sufficient amount of aligned reads for analyses.

### Differential Expression and Differential Splicing Analysis

In total, 110,982 protein coding isoforms were annotated, corresponding to 20,560 protein coding genes. After gene and isoform abundance filtering, 13,923 genes and 44,396 isoforms were selected for differential expression analysis at the gene- and isoform-level, respectively.

Although many known genes are differentially expressed, splicing variants can display a characteristic “switch” between major and minor isoforms. In addition to variations in overall gene expression, differential splicing may also be important to fully understand the underlying mechanisms involved in the pathobiology of IPF. Since differential splicing isoforms may play an important role in lung fibrosis [16], [20], we conducted differential splicing analysis at the whole transcriptome scale, investigating those genes in which the proportions of expressed isoforms change (major-minor isoform switch) between control and case conditions.

After abundance and variance filtering (Method Section), 3,098 genes with more than 1 expressed isoform were left as candidates for differential splicing analysis. Among these, 248 genes have Chi-square test False Discovery Rate (FDR) less than 0.05, and we considered these genes differentially spliced with statistical significance. The detailed information can be found in the Table S1.

### Joint Analysis of Differential Expression and Differential Splicing

**Figure 1:**
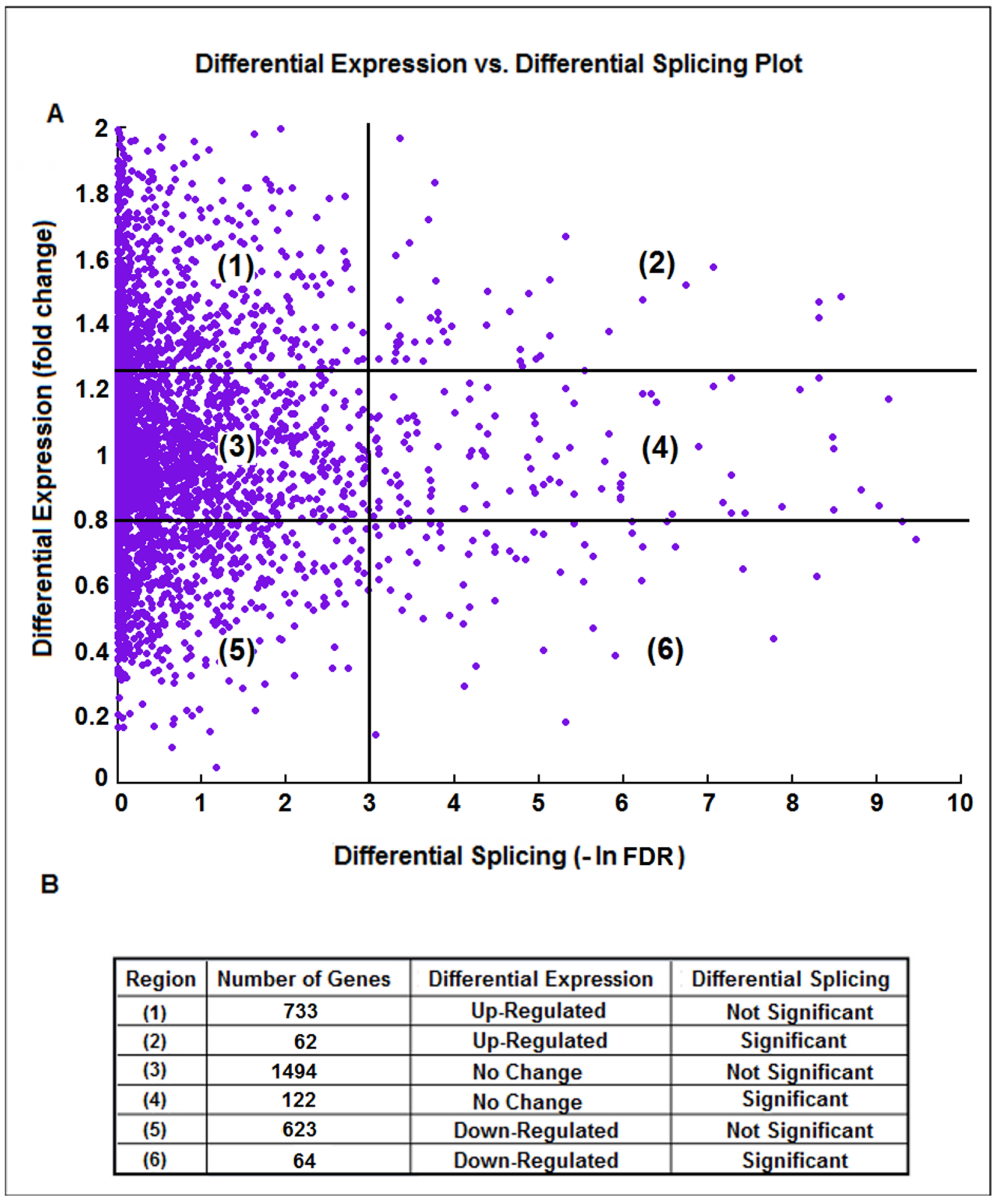
Joint analysis of differential expression and differential splicing between IPF lungs and controls. (A) Differential expression *vs*. Differential splicing plot. The panel is partitioned into 6 regions. (B) A table listing 6 regions representing the results of joint analysis of differential gene expression and differential splicing, and the number of genes within each region. Region 4 corresponds to the non-differentially expressed but differentially spliced genes that we carried out in-depth analysis.

Although newer microarray technologies, such as exon-junction array and tilting array, enable transcriptomic analysis at the isoform-level [31]–[34], the sensitivity and specificity are inherently limited by signal saturation, probe design and non-specific hybridization. Compared with microarray technologies, RNA-seq provides nucleotides sequencing at base-pair resolution, and therefore increases the accuracy of differential expression and differential splicing analyses. Since these two types of analyses examine different aspects of gene expression variation, it is necessary to perform a joint analysis to uncover novel biological events that could not be revealed by each alone. We attempted to identify IPF splicing variants that are consistent among replicates by examining the major-minor switch of isoform proportions within each gene. The combined information for these genes is presented in Figure 1.

In Figure 1A, 3,098 genes were plotted, with each purple dot corresponding to one gene, after abundance and variance filtering described in the Methods Section. The whole panel is further partitioned into 6 regions and detailed information is shown in Figure 1B. For each gene, we define up-regulation as fold change >1.25, down-regulation as fold change <0.8 and no change as fold change between 0.8 and 1.25. We also define significant differential splicing as –ln (FDR) >3 (corresponding to FDR value <0.05). In Figure 1A, most genes fall into region (1), (3) and (5), representing genes without major-minor isoform switches.

The genes located in regions (2), (4) and (6), however, are significantly differentially spliced, and would not be discovered by gene-level analysis. In particular, genes in region (4) are not differentially expressed at the gene level but display significantly differential splicing, so genes in this region represent a novel and previously uncharacterized region of regulation that warrants further investigation.

Here, we discuss in detail three examples (TOM1L1, CMTM4 and PEX11B) from region (4) (Figure 1A) with strong differential splicing evidence based on their read coverage signal maps. These cases correspond to three different types of alternative splicing mechanisms in IPF without significant changes at the gene-level.

**Figure 2:**
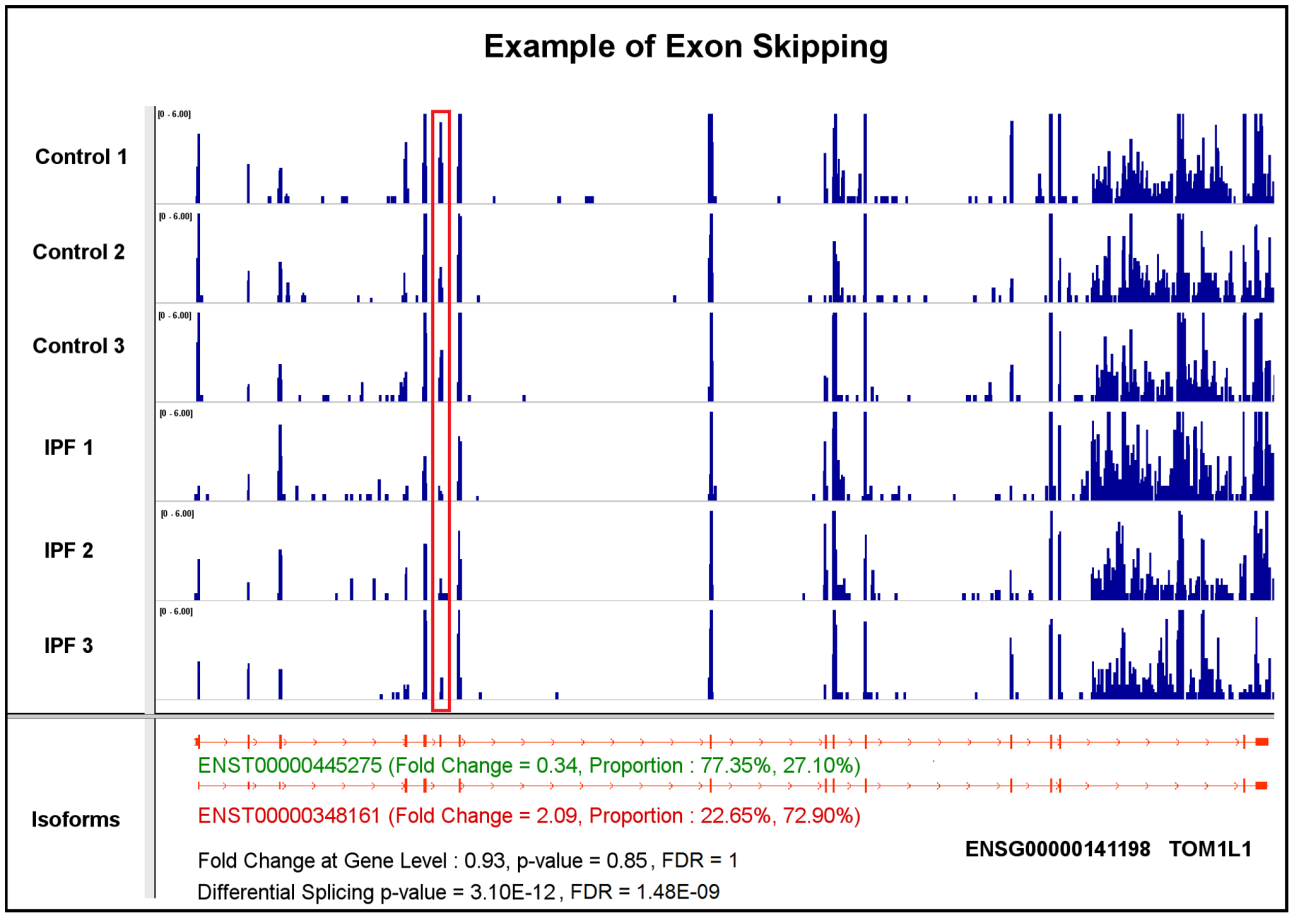
A case study of gene TOM1L1 illustrating the skipping exon splicing mechanism using the annotated transcripts.

**Figure 3:**
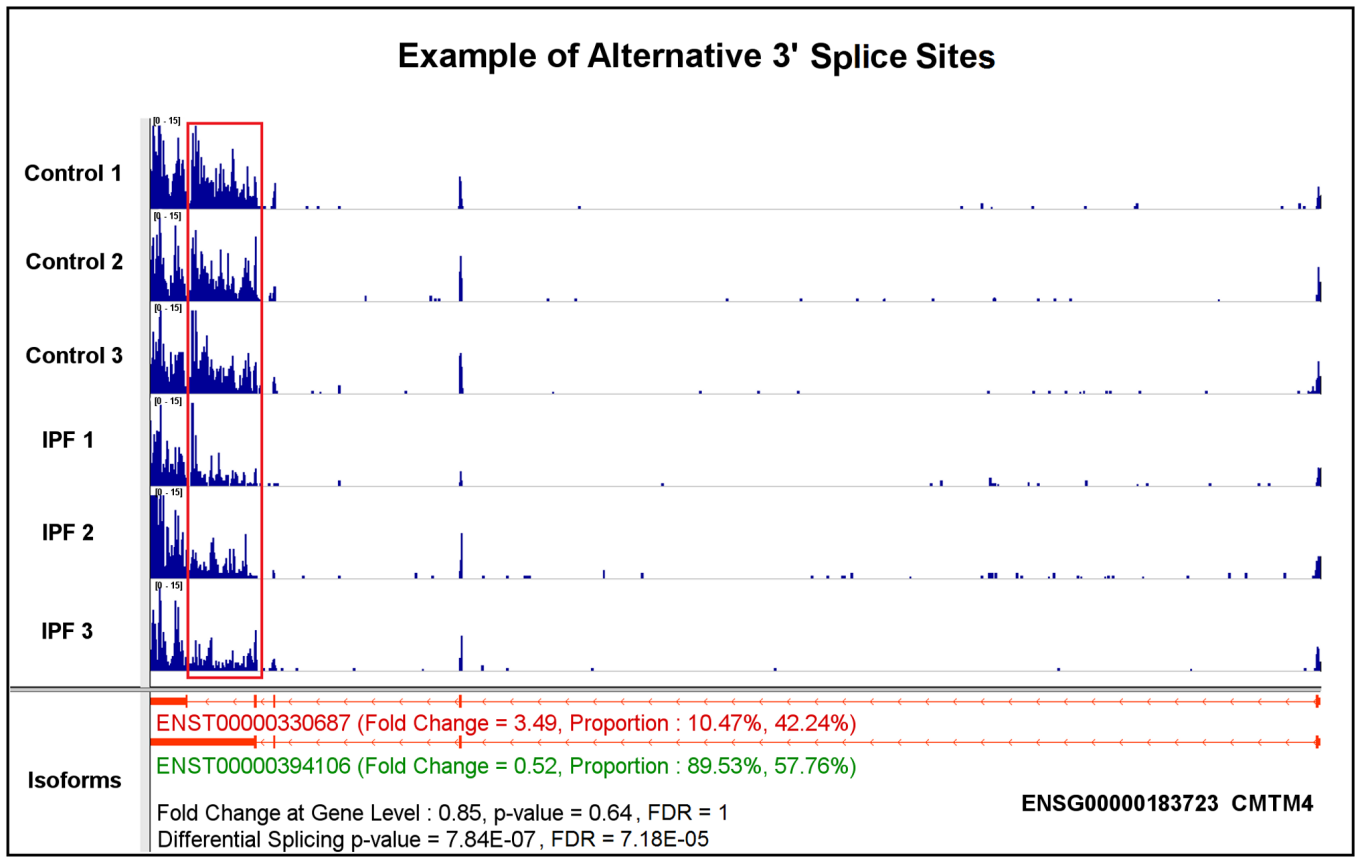
A case study of gene CMTM4 highlighting the alternative 3′ acceptor site splicing mechanism using the annotated transcripts.

**Figure 4:**
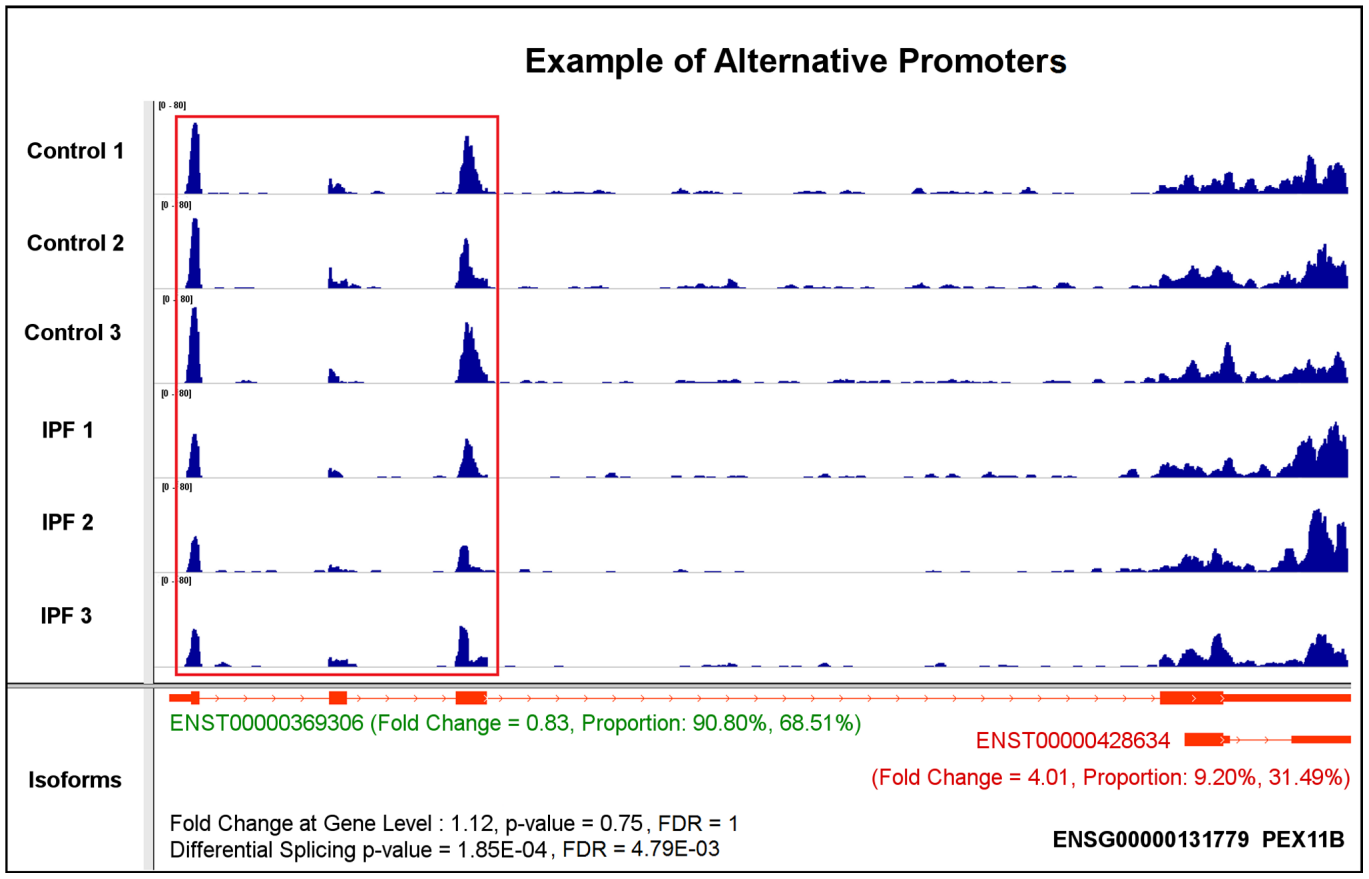
A case study of gene PEX11B as demonstration of the alternative promoters splicing mechanism using the annotated transcripts.

- (1).*Exon skipping or cassette exon*is the most common type of alternative splicing event in eukaryotic species [35]. A representative of this splicing mechanism is TOM1L1, which has two annotated isoforms: ENST00000445275 and ENST00000348161 (Figure 2). The major difference between these two isoforms is that the 6th exon of ENST00000445275 is skipped in ENST00000348161. Importantly, with a 0.93 fold change, the gene is considered to show no differential expression. However, based on our differential expression analysis at the isoform-level, ENST00000445275 is down-regulated and ENST00000348161 is up-regulated, with 0.34 and 2.09 fold changes, respectively. The observed gene-level differential expression ratio (DER) (0.93) represents the mixture of isoform-level DER (0.34 and 2.09). Meanwhile, the isoform proportion of ENST00000445275 decreases from 77.35% in control to 27.10% in IPF cases, while the isoform proportion of ENST00000348161 increases from 22.65% to 72.90%. These differences between control and case condition indicate a high degree of major-minor isoform switches, as the differential splicing FDR value is 1.48E-09. It also reveals the advantage of isoform-level differential expression and differential splicing analysis. The red box highlights the decreased read coverage at the skipped exon from control to case condition as the evidence of the exon skipping mechanism.
- (2). *Alternative 3′ splice sites* is another well-known alternative splicing mechanism [35]. Here, we present one of these two splicing events. CMTM4 has two expressed and annotated isoforms: ENST00000330687 and ENST00000394106 (Figure 3). The alternative 3′ acceptor site mechanism drives the difference between these two isoforms. There is no obvious differential expression at the gene-level as the fold change is 0.85. However, the calculated differential splicing FDR value is 7.18E-05, indicating proportional varieties at the isoform-level. For each individual isoform, ENST00000330687 is up-regulated by 3.49 fold change with this isoform proportion increasing from 10.47% in control to 42.24% in IPF, while ENST00000394106 is down-regulated by 0.52 fold with its proportion decreasing from 89.53% to 57.76%. Moreover, CMTM4 is also an example of the non-dominant isoform showing increases in both abundance level and proportion from control to case conditions, while the dominant isoform shows an opposite trend. As a result, the proportions of these two isoforms approach half-and-half in the diseased state. The red box highlights the change of read coverage signals as demonstration of this type of splicing mechanism.
- (3). *Alternative promoters* exist in more than half of human genes, which may vary the starting or termination site of transcripts for generating protein diversity [36]. As an example, two isoforms of gene PEX11B, ENST00000369306 and ENST00000428634, are expressed and annotated (Figure 4). The 5′-end truncated transcript ENST00000428634 is likely caused by an alternative promoter mechanism. Since the fold change at the gene-level is 1.12 and the differential splicing FDR value is 4.79E-03, this case also belongs to the category of no expression change at the gene-level, but significant changes in differential splicing. ENST00000428634 is significantly up-regulated about 4 fold with its proportion increasing from 9.20% in control to 31.49% in IPF. In contrast, the isoform proportion of ENST00000369306 decreases from 90.80% to 68.51%. The red box highlights the exons that are suppressed in IPF.

### Detection of Novel Transcripts

Although there are more than 100,000 isoforms annotated in splicing isoform databases, they are neither complete nor condition/tissue-specific. Because of this, the IPF-specific novel transcripts without annotation in splicing databases may play an important role in differentiating IPF from other interstitial lung diseases, or in helping researchers to better understand the pathogenesis of IPF.

We detected 1,479 novel junctions sharing exons with the annotated transcripts through skipping exon or mutually exclusive exon mechanisms, corresponding to a total of 1,252 genes. We composed 4,672 novel transcripts supported by these 1,479 novel junctions, and augmented the Ensembl database (version 60) by adding these novel transcripts. After joint analysis, we identified 35 genes that are differentially spliced and associated with novel transcripts with statistically significant FDR values <0.05. It is not known whether or not these genes and novel transcripts are relevant to fibrogenesis. The detailed information is available in the Tables S2 and S3. A case study was verified using qRT-PCR.

**Figure 5:**
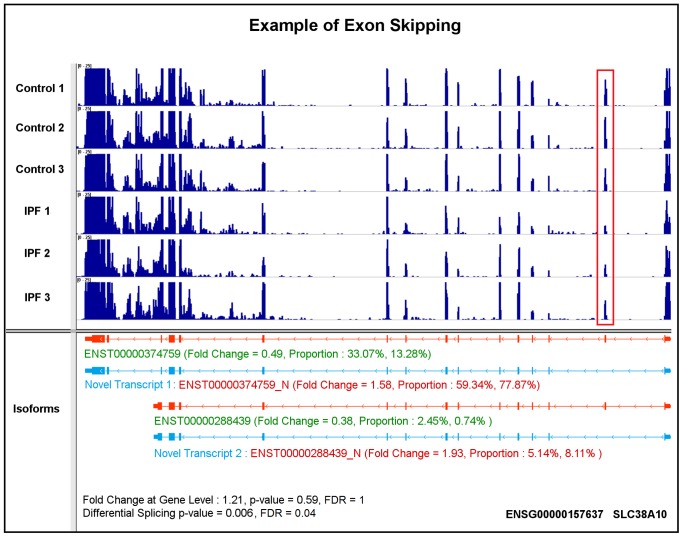
A case study of gene SLC38A10 illustrating the skipping exon splicing mechanism using both annotated and predicted transcripts.

Gene SLC38A10 encodes two novel transcripts with the skipping exon mechanism. As shown in Figure 5, SLC38A10 has two annotated isoforms, i.e., ENST00000374759 and ENST00000288439. With the novel junction evidence, we predicted that the novel transcripts, ENST00000374759_N and ENST00000288439_N (blue color in Figure 5), are associated with IPF. The two novel transcripts skip the second exon from their annotated ones (Figure 5). Analysis of the results shows that ENST00000374759 is down-regulated by 0.49 fold, and its isoform proportion is decreased from 33.07% in controls to 13.28% in IPFs, while ENST00000374759_N is up-regulated by 1.58 fold with proportion changing from 59.34% (in controls) to 77.87% (in IPFs). ENST00000288439 and ENST00000288439_N show the same trends with low abundance levels and proportions. The decreased read coverage at the skipped exon between the two conditions is highlighted within the red box.

### Quantitative RT-PCR

**Figure 6:**
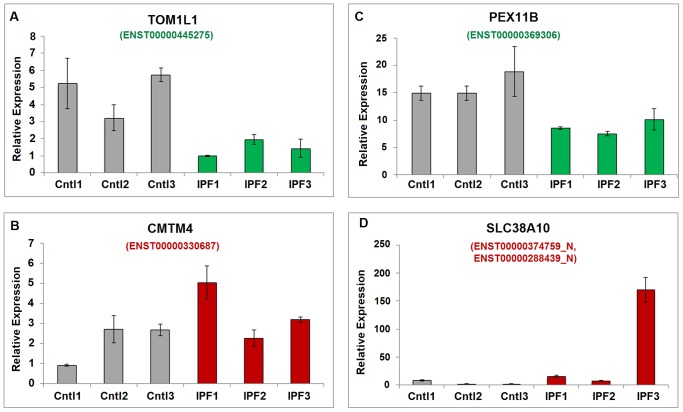
The qRT-PCR validation results for the annotated transcripts and the novel transcripts. (A) The validation result for the down-regulated (in green color) isoform ENST0000445275 of gene TOM1L1. (B) The validation result for the up-regulated (in red color) isoform ENST0000330687 of gene CMTM4. (C) The validation result for the down-regulated (in green color) isoform ENST0000369306 of gene PEX11B. (D) The validation result for the mixture of up-regulated (in red color) novel transcripts ENST00000374759_N and ENST00000288439_N of gene SLC38A10.

The predicted splicing variants were validated by quantitative RT-PCR analysis as shown in Figure 6, with the bar chart representing the relative expression values among three samples in each condition. One splicing variant was confirmed for each case study, and the experiment was performed in triplicate. In order to infer the up or down regulation of other splicing variants, we also quantified the common regions of the transcripts in genes TOM1L1 and PEX11B using qRT-PCR experiments. Due to the limited amount of sample tissues, we performed single validation experiment on each individual sample.

For TOM1L1, the PCR primers were designed at the region of the skipped exon. As shown in Figure 6A, the qRT-PCR analysis confirms the down-regulated expression of transcript ENST00000445275 in samples from IPF patients in a statistically significant manner (T-test *p*-value of 0.01). We further quantified the common region of the transcripts ENST00000445275 and ENST00000348161 in each individual sample tissue, and the results demonstrate a non-significant change (T-test *p*-value of 0.48) in expression abundance (Figure S1A). Collectively, we confirmed that the transcript ENST00000348161 is up-regulated as predicted.

In the case of CMTM4 (Figure 6B), the primers were designed at the junction region of the last two 3′-side exons of isoform ENST00000330687. The splicing variant ENST00000330687 was found to be up-regulated in IPF samples compared to controls. Although the difference doesn’t reach statistical significance, one IPF sample shows marked up-regulation of this transcript.

For gene PEX11B, the primers are located in the unique exon region of this isoform. The qRT-PCR data confirms a significant down-regulation of the ENST00000369306 isoform in the three IPF samples compared to control, as shown in Figure 6C, with a T-test *p*-value of 0.004. Similarly, in Figure S1B, the common region of both transcripts was quantified and no significant change (T-test *p*-value of 0.20) in relative expression among all the samples. Collectively, it confirmed that the transcript ENST00000428634 is up-regulated as predicted.

The presence of two new isoforms for SLC38A10, ENST00000374759_N and ENST00000288439_N, was confirmed by qRT-PCR. The PCR primers are designed at the new junction region of these predicted isoforms. The amplification product for the mixture of new transcripts was also analyzed for control and IPF samples, and the result is consistent with the predicted trend (Figure 6D).

## Discussion

Several array studies have been conducted to improve our understanding of the molecular processes involved in lung fibrogenesis, and to develop biomarkers. However, most of these studies are based on differential expression analysis at the gene level through microarray platforms. This type of analysis is a powerful tool in identifying gene patterns and pathways associated with IPF [37], [38]. Our extension of microarray discoveries was undertaken because recent publications on IPF indicate alternative splice variants occur in IPF and may be important for disease pathogenesis.

Splicing variants encoded by non-differentially expressed genes across conditions may play an important role in IPF. Thus, in this paper, we focused on detecting alternative splice variants from those non-differentially expressed genes, which have not been identified in previous pulmonary fibrosis microarray research. We applied abundance and variance filters at gene and isoform levels for detecting the most consistent splicing events in a conservative way. Our approach of joint analysis of differential expression and differential splicing appears to be useful in identifying splicing variants of IPF.

Protein variants as a result of alternative splicing have been shown to be associated with other human diseases. This has been exemplified in aging-related disorders, such as Alzheimer’s and Parkinson’s diseases, and involves aberrations in the alternative splicing of pre-mRNA. The incidence of idiopathic pulmonary fibrosis increases with age [39], though it is currently not clear if IPF is a disease of senescence or accumulated insult. Notably, it had been demonstrated that TGF-beta, an inducer of lung fibrogenesis, is capable of modulating the basic mechanism involved in alternative splicing of fibronectin [40], and that blocking the expression of the fibronectin EDA splice variant protects against lung fibrogenesis.

Changes in splicing could be a consequence of phosphorylated SF2/ASF, changes in other splicing factors or epigenetic changes in IPF lungs. The tissue-specific knockout of SF2/ASF revealed the disruption of only a subset of alternative splicing events, suggesting that a variation in concentration or activity of SF2/ASF is important for the activation of certain splicing events, the repression of others, and overall qualitative changes in alternative splicing patterns [41]. It is possible that some of the splicing variants identified in our study could be important in the pathogenesis and progression of this disease. But it is also likely that these genes are just a consequence of globally modified splicing regulation due to the activation of AKT/mTOR pathway [16].

DNA methylation changes and expression of chromatin modifiers could also be involved in the differential splicing observed in IPF. A recent study revealed an altered DNA methylation pattern in IPF with great similarity to the methylation pattern of lung cancer [42]. This is likely to be relevant for IPF, considering that several recent studies address the issue of non-promoter DNA methylation and the possible involvement in nucleosomal positioning and transcriptional regulation [43], [44]. We compared our analysis results with a recently published data on DNA methylation changes in introns and intron-exon-junctions in IPF lungs to normal lungs [42], and found that from 96 genes with significant changes at methylation in IPF, 3, 3 and 1 genes were located in regions (2), (4) and (6) respectively, defined as significantly differentially spliced in our model. We then correlated the methylation changes with the splicing variations in IPF for a subset of genes. Ninty-nine genes with changes in DNA methylation demonstrated no significant differential splicing in our system. However, 48 out of those 90 genes are in regions (1) and (5), which means that the gene expression or major isoform expression is altered, but no significant differential splicing was detected. The detailed information is available in the Table S4.

In addition, genes that are reported to show changes in methylation at the promoter region are not present in regions (2), (4) and (6). None of the reported hypermethylated genes showed significant change in differential splicing by this method, suggesting that hypomethylation in intragenic regions was mostly involved in the changes in splicing detected here. These results correlate with recent studies indicating that DNA methylation is substantially enriched at exons relative to introns, possibly for exon definition [45], [46]. Furthermore, several groups in recent years have found large-scale evidence for a link between nucleosome positioning and exon–intron architecture [47]–[52], thus pointing towards a connection between nucleosome positioning, DNA CpG methylation and differential splicing.

Aged lung has a predisposition for disrepair and for lung fibrosis [53], [54]. Recently, it has been shown that significant DNA methylation differences that account for changes in gene expression are associated with specific age-related disorders, and one of these genes is TOM1L1. TOM1L1 is known to be recruited to the endosome and can subsequently recruit clathrin. In addition, it has been reported that TOM1L1 is a regulating adaptor bridging activated EGFR with the endocytic machinery for internalization of activated EGFR [55]. Taking together, we can speculate that TOM1L1 could potentially serve as a marker for lung aging and maybe as a marker for susceptibility to lung fibrogenesis. However, we recognize that confirmation in a larger set of samples will be necessary to investigate this possibility.

Another gene detected by our method is CMTM4. The full-length cDNA product of CMTM4 is highly conserved during evolution [56]. CMTM4 is involved in cell growth and cell cycle regulation. The overexpression of CMTM4 can inhibit cell growth via G2/M phase accumulation [56]. It has been suggested that the deregulation of the transcription of CMTM4 in full-length (ENST00000330687) could contribute to stress-induced cellular senescence in epithelial cells that appears to be present in lung specimens from patients afflicted with IPF [57].

PEX11B is involved in peroxisome metabolic pathways and is likely to be involved in protecting the pulmonary epithelium against oxidative stress [58]. Recent reports also suggest that these and other related biochemical processes governed by this organelle play a critical role in regulating cellular aging. At low levels, peroxisomal ROS activates an anti-aging program in the cell, whereas at concentrations beyond a specific threshold, a pro-aging course is triggered [59]. PEX11B is present in all cell types in lung and have a conserved role in peroxisome maintenance through peroxisome proliferation, polarization, membrane elongation and segregation [58], [60]–[62]. Disruption of PEX11B results in a reduction in the total number of peroxisomes. A deficiency in the number and function of peroxisomes has been suggested to cause oxidative stress [63], [64]. This could be relevant, considering that oxidative stress is required for myofibroblast differentiation and it is hallmark of the IPF lung [65], [66]. Here we present “in silico” data for the differential splicing of this gene in IPF lung, and the experimental evidence that the transcript ENST00000369306 is down-regulated in IPF lung compared to normal lung. The confirmation of these data in an isolated set of samples will be important to confirm the relevance of PEX11B expression in the pathobiology of this deadly disease.

In addition to studying annotated transcripts, we also conducted an analysis of novel transcripts since the splicing variants associated with novel exon-exon junctions may reveal IPF-specific transcripts. The functional relevance of the novel transcripts for SLC38A10 will require further investigation.

Since our validated splicing variants were detected using a conservative approach, they may be useful in conjunction for the development of a biomarker that could be used to identify IPF, or active fibrogenesis. All the predicted regulatory trends of transcripts were consistent with the validation studies, although the validation results of two cases did not reach the common level of statistical significance. The number of samples and the low yield of RNA from each patient may limit the qRT-PCR validation in this study. In the future, more samples from IPF patients and control will be sequenced. Thereafter, a more comprehensive analysis and complete validation with quantification analysis of all isoforms for each gene of interest will be conducted for the discovery of potential isoform biomarkers for pulmonary fibrosis.

We speculate that the examples presented here reflect a generally modified state of the pre-mRNA processing machinery in IPF, leading to altered expression levels during aging or during accumulated lung insult. Similar analysis approaches may also be applicable to deciphering the pathobiology of other life-threatening diseases.

## Materials and Methods

### Sample Materials and RNA-Seq Data

Human IPF and control lung specimens were obtained from the NIH Lung Tissue Research Consortium (LTRC), and the detailed information about demographic and biological factors of IPF and control samples can be found in the Table S5. Total RNA was extracted from fresh frozen lung tissue using TRIzol Reagent (Invitrogen, Carlsbad, CA) following the vendor’s protocol. The RNA was deemed of high quality at Tulane University using the NanoDrop ND-1000 to assess the ratios of A260/A230 and A260/A280. RNA sequencing was performed at the National Center for Genome Resources, Santa Fe, New Mexico, where the total quantity of RNA was confirmed using the Qubit Fluorometer (Invitrogen, Carlsbad, CA), and RNA integrity was evaluated and found to be excellent using the Agilent 2100 BioAnalyzer Chip (Agilent Technologies, Santa Clara, CA) to assess the 18 s and 28 s bands to determine the extent of degradation. The transcriptomes of 3 IPF patient samples and 3 age-matched controls, defined as COPD with an FEV1>80% of predicted, were deep-sequenced using an Illumina Genome Analyzer II with a read length of 54 bases. This is considered a suitable control group because most patients with IPF have been smokers. For each tissue sample (biological replicate), over 25 million single-end reads were generated and stored in a file with fastq format. The RNA-seq data were submitted to the NCBI Short Read Archive with accession number SRA048904.

### Pre-processing and Alignment of RNA-seq Short Reads

We first performed a per base sequence quality check using fastQC Software (http://www.bioinformatics.bbsrc.ac.uk/projects/fastqc/). TopHat (v1.0.14) [67] was used thereafter to align short reads that were unique to the human reference genome (release hg19/GRCh37). Default settings and ‘–g 1′ parameter were used. Alignment results were saved in a SAM format.

### Expression Abundance Estimation at Both Gene and Isoform Level using SAMMate

Based on the alignment results of all the samples, expression abundance estimation was conducted at both gene and isoform levels using Ensembl ASTD database (version 60). SAMMate (http://sammate.sourceforge.net/), free Graphical User Interface (GUI) software, was employed for gene and isoform quantification. For isoform quantification, we applied the method, RAEM (Reads Assign by Expectation Maximization), reported in [24] and implemented in SAMMate. The output results of SAMMate contain not only expression abundance level measured by RPKM (Reads Per Kilobase of exon/transcript model per Million mapped reads) [68], but also aligned read counts for each gene and isoform, which were used as the input for differential expression analysis using edgeR [69].

### Differential Expression Analysis at Both Gene and Isoform Level

Firstly, an average RPKM cut off of 1 in both control and case conditions was applied to remove very low-abundance genes. And then, for those isoforms belonging to the remaining genes, we further filtered out very low-abundance isoforms, which have an average RPKM value less than 1 in both conditions. Finally, edgeR [69] was employed to prioritize the differentially expressed genes and isoforms with FDR values.

### Differential Splicing Analysis

We also applied RAEM to estimate isoform proportions for each gene. With the abovementioned abundance filtering results, in order to identify the most consistent differential splicing events, we also applied the variance filtering at the isoform-level. First, for each isoform of each gene, we multiplied the estimated isoform proportion by 100, e.g. enlarging 90% to 90, and then calculated the enlarged proportion variance for both control and case conditions. If the variance of enlarged proportion of every isoform in both conditions is smaller than 150 (the cutoff of the variance filter), we considered that the gene does not contain too much proportion variance on its isoforms, and keep these genes as candidates. Secondly, for each candidate gene, we constructed an n x 2 matrix. In the matrix, n rows correspond to the isoforms, and two columns correspond to the average of isoform enlarged proportions in control and case conditions respectively. Finally, for candidate genes, to detect differential splicing events between two conditions, we applied the Pearson’s Chi-squared test of independence (R function chisq.test) with Yates’ correction for continuity, and ranked those genes by FDR values, which are calculated from raw Chi-square *p*-values using Benjamini-Hochberg procedure [70].

In differential splicing analysis, we primarily focused on the divergence of isoform proportions for each gene across the IPF and control conditions. We examined all the genes as long as their expression abundances are above a certain threshold. In general, the Chi-squared test should be applied on actual count data, e.g. RPKM value of each isoform. However, directly using read count data has the following limitations: for highly expressed genes (>100 RPKM), even the minor change of isoform proportions between two conditions can yield significant differential splicing events (significant *p*-values); on the other hand, for relative low abundance genes (<10 RPKM), major change of isoform proportions between two conditions can be missed (non-significant *p*-values). Thus, we used the average of enlarged proportions as pseudo counts to make the *p*-values comparable across the genes of different abundances and achieve a more robust detection of differential splicing.

### Detection of Novel Transcripts

Firstly, we constructed novel transcripts supported by the IPF-specific exon-exon junction evidence form TopHat [67], i.e. specific to the IPF splicing events, using the same method showed in Figure 1b from Deng et al. [24]. Then, we augmented the Ensembl database (Homo Sapiens.GRCh37.60) by adding novel transcripts. Thereafter, we conducted the isoform quantification analysis, followed by the joint analysis of differential expression and differential splicing with abundance and variance filtering to detect the IPF-specific novel transcripts from non-differentially expressed genes.

### Visualization of Case Studies

The case studies were explored using Integrative Genomics Viewer (IGV) [71] (version 1.5), free software available from www.broadinstitute.org/igv. To visualize the read coverage signal map of control and IPF samples in IGV, the wig files generated from SAMMate were converted to the compatible IGV format using the “Tile” function of igvtools. The igvtools were embedded in the IGV software.

### Quantitative Real-time PCR

The analysis of differential expression of transcripts was conducted using quantitative real-time PCR. The samples are the same for RNA-seq. One ug of RNA was used for reverse transcription by using iScript cDNA Synthesis Kit (Bio-Rad, Hercules, CA) according to the manufacturer’s protocol. Quantitative real-time PCR was employed to measure RNA expression using IQTM SYBR Green super mix (Bio-Rad, Hercules, CA). Primers were designed using Primer-Blast software from NCBI. Expression data were normalized to the housekeeping gene 36B4, which has been established in the laboratory for decades as a reference gene for IPF studies, using the 2?-deltadeltaCt method described by Livak and Schmittgen [72]. For all genes, the presence of a single amplification product was verified by analysis of melting curves of reverse transcription-PCR reaction. The cases of TOM1L1, PEX11B, CMTM4 and novel transcripts of SLC38A10 were validated by qRT-PCR using primers indicated in Table S6.

## Supporting Information

Figure S1 **The qRT-PCR validation results of the common regions of transcripts in gene TOM1L1 and PEX11B.**
(TIF)

Table S1 **Analysis results of differential expression and differential splicing of 3,098 annotated genes.**
(XLS)

Table S2 **Annotation of 35 genes with annotated and novel transcripts.**
(XLS)

Table S3 **Analysis results of differential expression and differential splicing of 35 genes with novel transcripts.**
(XLS)

Table S4 **Comparison results between our study and a published dataset.**
(XLS)

Table S5 **Demographic and biological factors of IPF and control samples.**
(XLS)

Table S6 **Primers used in qRT-PCR validation study.**
(XLS)
