# Supplementary figures and images for: Detecting Splicing Variants in Idiopathic Pulmonary Fibrosis from Non-Differentially Expressed Genes
Source: PLoS One. 2013 Jul 2;8(7):e68352. doi: 10.1371/journal.pone.0068352 (PMC3699530; doi:10.1371/journal.pone.0068352)

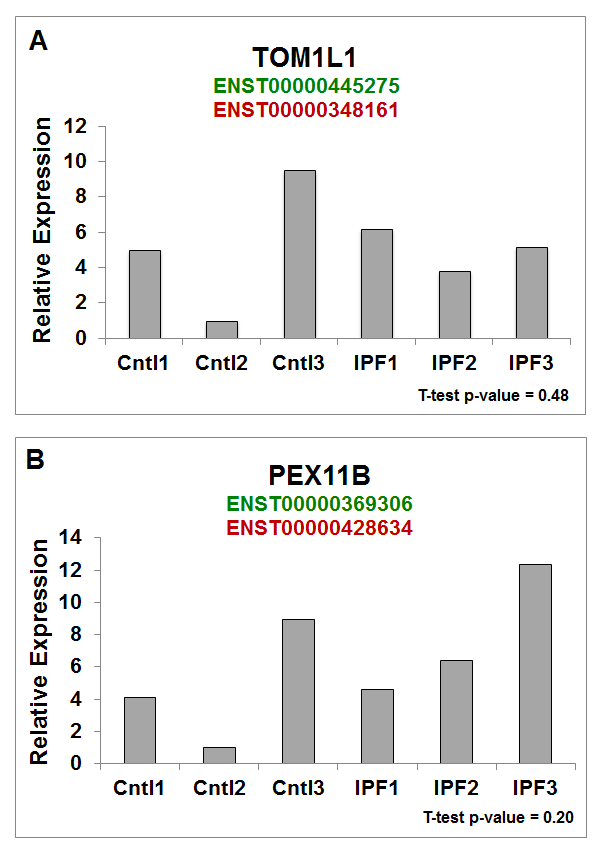

Supplement: Figure S1 — The qRT-PCR validation results of the common regions of transcripts in gene TOM1L1 and PEX11B. (TIF) [file pone.0068352.s001.tif]
